# Exploring the Emotional Experiences of First-Time Fathers During Infancy: A Qualitative Study

**DOI:** 10.1177/15579883261445266

**Published:** 2026-05-23

**Authors:** Sharanya Chatterjee, Vaishalee Saravanan, Samyukta Vaidyanathan

**Affiliations:** 1 Department of Clinical Psychology, Manipal College of Health Professions, Manipal Academy of Higher Education, India

**Keywords:** first-time fathers, paternal mental health, fatherhood, men’s health, infancy, qualitative research

## Abstract

Becoming a first-time father is a profound experience that shapes a man’s emotional and psychological well-being. While the role of mothers during infancy has been extensively explored, the emotional experiences of first-time fathers remain powerful yet often overlooked, leaving gaps in understanding their challenges, support needs, and mental health implications. The study employed a qualitative, exploratory research design. In-depth semi-structured interviews were conducted with 13 first-time fathers, recruited via purposive sampling. Data were analysed using thematic analysis to explore the emotional landscape of first-time fathers during infancy. The analysis yielded 20 sub-themes, organized under five overarching themes. These themes encompassed paternal concerns for maternal well-being, fatherhood-related transformations, positive experiences of fatherhood, adjusting to infant care, and responsibilities and support systems. The findings indicate that the journey of first-time fatherhood is dynamic, characterized by significant emotional changes, shifting identities, and the ongoing balancing act between responsibility and joy, with supportive relationships playing a vital role in shaping their experiences. The study contributes to a broader understanding of fatherhood by providing insights into the emotional terrain first-time fathers navigate, with implications for clinical practice, policy development, and future research in paternal mental health. By giving voice to an often-silenced journey, this study highlights the need for greater recognition and support of fathers during this critical life stage.

## Introduction

A first-time father is defined as a “biological father who lived with the expectant mother during pregnancy and has not experienced the live birth of his own child” (Chandler & Field, 1997). This stage represents a significant life transition beginning during pregnancy and continuing through the first year of the child’s life.

### Transition to Fatherhood

The transition is described as a “developmental engine” that acts as a catalyst for personal growth during the prenatal period (Carlson et al., 2015; Walsh, 2014).

Galinsky’s (1987) “Stages of Parenthood” highlights the emotional shifts experienced by first-time fathers during infancy. In the initial “Image-Making Stage” (pregnancy to infancy), fathers begin forming their parental identity by developing expectations, beliefs, and emotional bonds with their unborn and newborn children. This stage involves psychological recalibration as fathers navigate new responsibilities, changing relationships, and societal expectations. Assistance during this period helps fathers strengthen bonds with their infants, develop their sense of self, and cultivate generative skills.

### Fathers’ Role During Pregnancy and Childbirth

Fathers play an important role during pregnancy and childbirth by providing emotional, practical, and informational support. Active paternal involvement has been associated with reduced maternal stress, improved pain management, and better birth outcomes and long-term maternal well-being (Hasman et al., 2014; Kaye et al., 2014). Fathers’ presence and help during labour can aid women feel more prepared and may contribute to shorter labour and improved maternal health outcomes (He et al., 2015; Kaye et al., 2014; Longworth & Kingdon, 2011; Steen et al., 2012). Research suggests that paternal involvement may reduce risks such as preterm birth, low birth weight, and intrauterine complications (Bäckström & Hertfelt Wahn, 2011; Premberg et al., 2011).

First-time fathers frequently report fear, anxiety, helplessness, and uncertainty, particularly due to limited information and unfamiliarity with the birthing process (Koyuncu & Kıssal, 2025; Labrague, 2013; Poh et al., 2014). Concerns about their partner’s pain and the baby’s health further intensify these emotions (Hildingsson et al., 2011). Despite these challenges, fathers often describe a shift from fear to confidence and happiness following childbirth. Limited inclusion of fathers in delivery settings and inadequate preparation can increase anxiety, highlighting the need for father-inclusive health care practices (Koyuncu & Kıssal, 2025; Smith et al., 2024). Fathers may contribute practically during pregnancy by attending prenatal appointments, assisting with household responsibilities, participating in health care decisions, and reassuring their partners during labour and delivery.

### Emotional and Psychological Changes

First-time fathers experience a complex mix of excitement, anxiety, and responsibility, with emotions ranging from stress and uncertainty to joy, pride, and fulfilment (Eggermont et al., 2017; Koyuncu & Kıssal, 2025; Sapountzi-Krepia et al., 2010). Role shifts, caregiving responsibilities, and witnessing their partner’s physical and emotional changes often deepen fathers’ emotional connection and sense of responsibility (Hodgson et al., 2021; Talley, 2017).

These emotional responses are accompanied by psychological adjustments as fathers redefine their priorities and responsibilities within the family. Studies indicate that during the first 1,000 days fathers experience mixed emotions such as love, pride, excitement, anxiety, and self-doubt (Vidinha et al., 2025). Fathers may suppress their emotions due to societal expectations discouraging vulnerability (Sapountzi-Krepia et al., 2010). Several theoretical frameworks help explain fathers’ adaptability during this transition, including Belsky’s (1984) Transition to Parenthood Theory, Bowen’s (1978) Family Systems Theory, and Lazarus and Folkman’s (1984) Stress and Coping Theory. Fathers may cope by seeking guidance from health care professionals, sharing responsibilities with partners, and engaging in peer support. Alongside stress and ambivalence, many fathers report personal growth, increased sensitivity, and shifts in priorities and identity (Škvařil & Presslerová, 2024; Smith et al., 2024).

### Social Support and Health Care Involvement

Social support and health care involvement play an important role in facilitating fathers’ integration to parenthood. Support from partners, extended family, domestic help, and health care professionals can reduce stress, increase confidence, and strengthen paternal engagement (Smith et al., 2024; Vidinha et al., 2025). Barriers such as nuclear family structures, financial pressures, and limited father-specific services may hinder involvement. Research highlights gaps in mental health support for fathers during the perinatal period, with many experiencing exclusions from health care services primarily designed for mothers and facing barriers in seeking help due to masculine norms and self-stigma (Reay et al., 2024). Workplace demands and limited father-focused health care may restrict paternal involvement despite the benefits of active participation for family well-being. Inclusive health care practices and supportive workplace policies, including paternity leave, are important for improving paternal mental health and family outcomes (Hodgson et al., 2023; Leahy-Warren et al., 2022; Smith et al., 2024).

### Cultural Context in India

Fatherhood is shaped by cultural and regional contexts. In India, fathers are traditionally viewed as financial providers, while caregiving responsibilities are largely assigned to mothers. Participation in prenatal and postnatal care may be shaped by societal norms, rigid gender roles, and institutional practices (Ganapathy, 2016; Premberg et al., 2011).

These experiences vary across regions, with rural fathers often expected to focus primarily on earning income, while urban fathers may have more opportunities for involvement in prenatal care. Socio-economic status, caste, and education further influence fathers’ engagement and emotional experiences. Masculinity is closely tied to fatherhood in many contexts, creating pressures that may lead to emotional suppression and role conflict. Role Theory (Biddle, 1986) helps explain how fathers navigate tensions between societal expectations and personal involvement in parenting. In many Indian households, extended family members play an active role in childcare and decision-making during the perinatal period. This intergenerational involvement can shape fathers’ participation in caregiving and influence how parenting responsibilities are distributed within families.

This study explores the emotional, social, and cultural experiences of first-time fathers during infancy. By examining fathers’ challenges, coping strategies, and positive experiences, the study contributes to a deeper understanding of paternal involvement during the early stages of parenthood within the Indian context. Recognizing fathers’ strengths and challenges may help inform efforts to enhance paternal mental health, strengthen parent–infant bonding, and promote healthier family dynamics.

## Method

A qualitative exploratory approach was used to explore the experiences of first-time fathers during infancy, guided by the following research question: What are the experiences of first-time fathers during the initial period of fatherhood?

### Study Design and Setting

A qualitative exploratory design was used to understand first-time fathers’ experiences during infancy. Data was gathered through in-depth semi-structured interviews, which were subsequently analysed using thematic analysis (Braun & Clarke, 2006) to uncover significant themes and sub-themes. The study spanned one and a half years, from October 2023 to May 2025 at the Department of Clinical Psychology at Manipal College of Health Professions, Manipal. All interviews were conducted via secure online platform Microsoft Teams, audio-recorded, and no third parties were present. Online interviews were conducted to reach a wider sample across various regions of India (Supplemental Material).

### Participants and Sampling Procedure

The research focused on first-time fathers aged between 25 and 45 years who lived with their infant (aged 1 year or younger) and the mother of the baby. Participants needed to speak English, have internet access, and be open to sharing their emotional experiences related to pregnancy and the early stages of fatherhood. To qualify for the study, the infant had to be full-term (born at 37 weeks’ gestation or beyond) and weigh at least 2,500 g at birth. English-speaking participants were selected due to the interviewer’s language proficiency and to ensure accuracy in data interpretation.

Fathers with severe mental health issues, who were divorced or separated, stepfathers, and those with children suffering from chronic health conditions or congenital anomalies were not included. Participants were approached through the hospital outpatient departments and via online flyers on parenting forums and social media. Interested participants contacted the researcher directly. Purposive sampling was used to recruit participants who could provide rich, detailed narratives of first-time fatherhood experiences during infancy. A total of 13 participants were approached and all agreed to participate. Data collection continued until data saturation was achieved at the 13th interview, defined as the point when no new themes emerged. Demographic characteristics of participants were recorded and summarized to contextualize findings.

### Researcher Characteristics, Reflexivity, and Credibility

Interviews were conducted by the first author, a female clinical psychology post graduate trainee. The researcher’s background and experiences as a mental health professional informed the interview process and data interpretation. The interviewer was trained and certified in qualitative research. No prior relationship existed between the researcher and participants. Participants were informed about the researcher’s role and study purpose.

To minimize bias, reflexive bracketing was employed, involving continuous self-reflection and journaling to identify and set aside personal assumptions. The researcher acknowledged assumptions related to fatherhood expectations and actively reflected on them during journaling. The research team included members with academic, clinical, and experiential perspectives, providing both insider and outsider viewpoints that enhanced interpretation of cultural, emotional, and gendered aspects of early fatherhood.

Credibility was enhanced through peer debriefing, reflexive journaling, and an audit trail documenting decisions throughout the research process. Data triangulation was not performed because the study focused solely on in-depth interviews. Credibility was instead strengthened through peer debriefing, an audit trail, and member checking. The thematic analysis was conducted using a constructivist perspective to authentically reflect participants’ narratives.

### Data Collection Procedure

Ethical clearance was obtained from the Department of Clinical Psychology, followed by approval from the Institutional Research Committee and the Institutional Ethics Committee. The study was registered with the Clinical Trials Registry of India (CTRI).

A standardized socio-demographic and clinical data sheet was employed to gather pertinent participant information. Following recruitment, rapport was established with each participant, and data were gathered using a validated semi-structured interview schedule that included eight to nine open-ended questions. The interview included questions about emotional experiences during pregnancy, childbirth, infant care, role transitions, mental health, and support systems. The interview schedule was validated by a panel of five professionals, including a clinical psychologist, psychiatrist, neonatologist, perinatal mental health specialist, and qualitative research professional. The interview guide was pilot tested with two participants and refined based on feedback. Interviews were conducted by the first author (clinical psychology trainee). Each interview was conducted once and lasted 50 to 60 minutes. Field notes were taken after each session. All sessions were recorded with consent, anonymized, and securely stored.

### Data Analysis and Coding

The data were verbatim transcribed and analysed using Braun and Clarke’s (2006) six-phase thematic analysis approach, which includes (1) to ensure familiarity with the content, the interviews were transcribed verbatim, and the transcripts were read and reread while noting initial thoughts and reflections. (2) Important data features were coded line by line methodically. To find recurring patterns, these codes were arranged in an Excel document for every participant. These codes were then colour-coded and categorized into new sub-themes. (3) Expanding on this, the sub-themes were grouped into more overarching themes, with consideration being given to their relationships. (4) Themes were then thoroughly examined in relation to the coded extracts and the entire dataset. To ensure internal consistency, themes were merged, redefined, or eliminated. (5) After they were completed, the themes and sub-themes were distinguished and given succinct, illustrative names. (6) Producing the final report which included selecting representative excerpts to highlight each topic and developing the findings into an analytical narrative that met the goals of the study and was interpreted in light of previous research. Coding was done manually with no software utilized. A coding tree was developed and maintained in Excel. After initial coding, a code list was generated, and similar codes were grouped into sub-themes and themes. A preliminary codebook developed by the researcher was independently reviewed by two researchers. Themes were then refined and validated against the full dataset to ensure accuracy, and recurring patterns were consolidated. The research guide and co-guide reviewed and validated the final codebook to ensure it met standards of rigour and reliability. Intercoder agreement was reached through iterative discussion until consensus was obtained. Transcripts were returned to participants for verification and member checking was conducted with four participants to confirm the accuracy of the themes and feedback was incorporated by revising theme labels and merging overlapping themes.

### Ethical Considerations

The research was approved by the Institutional Ethics Committee, Kasturba Medical College and Kasturba Hospital, Manipal (IEC2 448/2024), and officially registered with the Clinical Trials Registry—India (CTRI/2024/09/074473). Participants were provided with a participant information sheet that had all the details of the study after which they provided informed written consent, ensuring confidentiality, the choice for voluntary involvement, and the right to withdraw at any time. A socio-demographic data sheet and clinical data sheet were also collected from the participants. No compensation was provided. Interviews took place on secure online platforms and were recorded with participants’ consent. Data were anonymized and kept in a password-protected system by the Principal Investigator, with plans to retain it for 3 years following the study completion before secure deletion.

## Findings

The demographic and clinical details of the participants are presented in Table 1. The mean age of the fathers was 34 years. Of the 13 participants, 15% held a PhD, 54% had completed a master’s degree, 23% had a bachelor’s degree, and 8% possessed a diploma. Participants came from various professional fields, including banking, business, education, and real estate; 23% were IT professionals, and one participant was unemployed at the time. The majority of participants did not report any major physical health issues, although one father had been diagnosed with papillary carcinoma of the thyroid.

**Table 1.** Demographic Data of Participants (n = 13).

Participants	Age (in years)	Educational qualification	Occupation	Presence of any physical health issues
P1	33	Bachelor of Technology	IT professional	No
P2	34	Master of technology	Bank Manager	No
P3	33	Master of Computer Applications	IT professional	No
P4	32	Master of Science	Branch Manager	No
P5	34	Master of Business Administration	Self-Employed	No
P6	33	Bachelor of Commerce	Real Estate Agent	No
P7	37	Bachelor of Technology	Unemployed	No
P8	34	Doctor of Philosophy	Assistant Professor	No
P9	41	Doctor of Philosophy	Scientist	No
P10	32	Master of Science	Software Engineer	No
P11	36	Diploma in Engineering	College Staff member	No
P12	35	Postgraduate	Business	No
P13	33	Master of Science in Zoology	Medical Representative	Yes

During the data analysis, a total of 1,153 codes were generated. These codes were systematically refined into 20 sub-themes and further organized into five main themes: (1) Paternal Concerns for Maternal Well-being, (2) Fatherhood Transformations, (3) Positive Experiences of Fatherhood, (4) Adjusting to Infant Care and Responsibilities, and (5) Support Systems, as presented in Table 2.

**Table 2.** Themes and Corresponding Sub-themes.

Themes	Sub-themes
1. Paternal Concerns for Maternal Well-Being	1.1 Pregnancy-related concerns
	1.2 Challenges during delivery
	1.3 Wife’s postnatal experiences
	1.4 Superstitious beliefs
2. Fatherhood Transformations	2.1 Gender expectations 2.2 Planning and initial preparations
	2.3 Mixed feelings
	2.4 Growing responsibility and paternal evolution
	2.5 Shared parental experiences
3. Positive Experiences of Fatherhood	3.1 Initial responses upon hearing the news
	3.2 Feelings as a new father
	3.3 Bonding with the baby
	3.4 Memorable moments
4. Adjusting to Infant Care and Responsibilities	4.1 Early caregiving challenges
	4.2 Infant health and paternal soothing
	4.3 Work–family balance
5. Support Systems	5.1 Support and reassurance for partner
	5.2 Grandparents’ support
	5.3 Availability of house help
	5.4 Professional and social support

### Theme 1: Paternal Concerns for Maternal Well-Being

This theme explores the role of the husband in ensuring the well-being of their spouse during pregnancy and early parenthood. Fathers described feeling a deep sense of responsibility for providing emotional, physical, and hands-on support to their wives. This theme comprised four sub-themes: *Pregnancy-related concerns, Challenges during delivery, Wife’s postnatal experiences, and Superstitious beliefs.* Their concerns ranged from their partner’s health and nutrition to managing household responsibilities and ensuring a stress-free environment. Seventy-seven percent of fathers (*n* = 10/13) expressed a strong protective instinct, constantly monitoring their wife’s safety and making necessary alterations to their daily lives to accommodate her needs.

I was only thinking about keeping my wife properly. Yeah, I was very anxious about one thing that she should not fall, at the bathroom or at any door, at some places of our home, there is some bump on there, So I was very anxious about those things and also with the health nutrition I was concerned about all those things and I was taking care of all those things. (P7)

First-time fathers experienced a range of emotional hurdles and engaged actively in caregiving during pregnancy. In the sub-theme *1.1 Pregnancy-related concerns*, approximately 23% of fathers (*n* = 3/13) expressed persistent anxiety stemming from prior miscarriages, which affected their cautious approach to family planning and postponed announcements of the pregnancy, like one of the participants expressed, “She didn’t want to check. She was too scared because of what had happened before. I told her we should wait and be sure before telling anyone” (P8). Eighty-four percent of fathers (*n* = 11/13) prioritized the health and well-being of their wives, frequently taking on household duties and making personal sacrifices to offer support. Alternatively, 54% of fathers (*n* = 7/13) reporting uncomplicated pregnancies while 31% (*n* = 4/13) had encountered medical issues, leading fathers to become heavily involved in managing symptoms like nausea, cravings, and mood swings, often assuming extra responsibilities and providing emotional assistance.

The delivery and postnatal periods were marked by intense emotional experiences and caregiving demands. In the sub-theme *1.2 Challenges during delivery*, the delivery process, particularly in cases of emergency C-section, 23% of fathers (*n* = 3/13), frequently proved to be unpredictable and stressful, leaving fathers feeling both helpless and committed to support their partners. The sub-theme *1.3 Wife’s postnatal experiences* talks about postpartum challenges, which affected some of the mothers, involving issues such as haemorrhage, recovery from surgery, and challenges with breastfeeding, which necessitated continuous physical and emotional care from fathers, like one of the fathers stated, “She had a lot of pain due to the back anaesthesia. It started spreading to her head, and at one point, she couldn’t tolerate it, she was crying. The doctor suggested complete rest for her” (P8). Despite these hardships, fathers remained devoted caregivers, acknowledging their partners’ resilience and focusing on the well-being of their family. The joy of welcoming their newborn often overshadowed the stress and exhaustion that accompanied childbirth and recovery.

Cultural expectations and *Superstitious beliefs—*sub-theme 1.4 shaped the experiences of 31% of fathers during pregnancy and childbirth, often clashing with contemporary medical advice. While certain fathers found it challenging to resist traditional customs, the majority actively chose to discard outdated practices in favour of ensuring the well-being of their wives and babies. Being physically distant from extended family enabled some fathers to sidestep the pressure to adhere to cultural norms. Notably, 92% of the fathers (*n* = 12/13) exhibited a strong confidence in modern medicine, indicating a trend towards prioritizing evidence-based care over cultural superstitions.

### Theme 2: Fatherhood Transformations

From early preparation and conflicted feelings to accepting complete responsibility, this theme examines the life-changing experience of parenthood. This theme comprised five sub-themes: *Gender expectations, Planning and initial preparations, Mixed feelings, Growing responsibility and paternal evolution, and Shared parental experiences.* As they negotiated their roles as parents, fathers talked about their changing priorities, lifestyle changes, and personal development. Their experiences demonstrate how their journey into fatherhood was influenced by the practical, financial, and emotional preparations they made.

Becoming a father changed me in ways I didn’t expect. My priorities shifted completely, and everything started revolving around my child. (P7)

First-time fathers encountered a complicated emotional journey during the planning and initial phases of becoming parents. The sub-theme *2.1 Gender Expectations* talked about how fathers dealt with complex gender norms, with 31% of fathers (*n* = 4/13) showing a preference for daughters and 23% (*n* = 3/13) experiencing family pressure for sons, although most emphasized the importance of the child’s health over gender.

I’ve heard about people saying that I wouldn’t, . . . that I haven’t handled the baby, and yeah, those were as main due to stereotypes, the people didn’t believe that, men were capable of handling babies, or it was mainly a gender role thing where they believed that a mother should be nursing, and taking care of the baby when very young, but I, from the very beginning I didn’t believe in that, and also I think taking care of the baby builds the bond between me and the baby, and I love doing everything for him. (P9)

In the sub-theme *2.2 Planning and initial preparations*, 69% of fathers (*n* = 9/13) actively prepared for pregnancy, incorporating financial stability, health care decisions, and long-term family objectives into their choices. Many underscored the necessity of ensuring their child’s future through investments, insurance, and saving for education. Despite this preparation, 54% of them (*n* = 7/13) experienced *Mixed Feelings* sub-theme 2.3 comprising excitement and anxiety, struggling with self-doubt and fear of the unknown, especially during early medical check-ups, as one participant noted, “Family members didn’t know until two months from at that point, we didn’t want to disclose until the first USG, that’s until the pregnancy is confirmed” (P9). Societal expectations and traditional norms appeared to influence how some fathers expressed their emotions, while some faced extra difficulties, such as personal health challenges, which made the transition even harder. Nevertheless, fathers slowly started to reconcile their fears with an increasing sense of responsibility and optimism.

I mean, coming from an Indian family, from a, you know, a conservative Indian family, the only thing you want is you want a good value system and a good education for your child, right? You just want to teach your child to be good and have good values. So that’s mostly what I really wanted to give my child, or I still want to give that to my child. (P10)

Fatherhood instilled a heightened *Growing responsibility and paternal evolution—*sub-theme 2.4; as 54% of fathers (*n* = 7/13) willingly took on a proactive role in meeting emotional, financial, and practical needs for their families. This phase was characterized by notable personal development and changes in lifestyle, 85% of fathers (*n* = 11/13) indicated they had grown in maturity, realigning their priorities to concentrate on their infant’s needs rather than their own personal or career goals. Fathers assumed new responsibilities such as household tasks and caregiving, which helped them cultivate greater patience and resilience. Notably, 69% (*n* = 9/13) regarded parenting as a collaborative journey with their partner, highlighting the importance of teamwork in areas like childcare, medical appointments, and financial planning as outlined by the sub-theme *2.5 Shared parental experiences.* One of the participants expressed, “My wife and I handled everything as a team, from sleepless nights to hospital visits. It was never just one person’s responsibility” (P1). They intentionally adjusted their schedules and responsibilities to foster a stable and nurturing environment, demonstrating a joint effort in parenting and a dedication to their family’s welfare.

My lifestyle has changed a bit obviously because I’ll tell you when in the month of April when she was born then one month gap was there, I was alone . . . so in normal course of time I would wake up at 8.30, but at that point of time, my wife told me “you’ve to prepare this food for the baby since she was on different diet,” so I would’ve to wake up at 7:30 or at least 8:00, half an hour before and I had to do the dishes and I had to cut the vegetables for her, whatever doctor prescribed. I figured out I have never done this for the remainder of my life before this period, so now suddenly when she’s there and I have to take care of her, suddenly, my life has already started changing. Also, I used to come back from the office and cook so now there is one more member, it feels good for me. (P2)

### Theme 3: Positive Experiences of Fatherhood

The happiness, contentment, and strong emotional ties that fathers developed throughout pregnancy and the early years of parenthood are encapsulated in this topic. This theme comprised four sub-themes: *Initial responses upon hearing the news, Feelings as a new father, Bonding with the baby, and Memorable moments*. Despite the challenges of early fatherhood, many fathers described deeply joyful and emotional moments such as learning about the pregnancy, attending ultrasounds, and holding their baby for the first time as life changing. These experiences brought pride, love, and purpose while strengthening family bonds, with relatives sharing in the joy of the new arrival. These treasured moments stood out as turning points that fathers would remember for the rest of their lives, despite the worries that came with pregnancy and childbirth.

It was exciting. I mean, it was very exciting. We were kind of, you know, we were prepared for it. It was not like we were not. We were kind of also planning for it and all that. So, it was. It was exciting. We were very happy. (P10)

Sixty-nine percent of the first-time fathers (*n* = 9/13) felt overwhelming joy, while 38% (*n* = 5/13) exhibited a more subdued happiness as their *Initial responses upon hearing the news—*sub-theme 3.1. This news was frequently greeted with excitement by extended family members, strengthening connections and prompting immediate preparations. For numerous fathers, this moment signalled the beginning of a deeply satisfying journey filled with a strong sense of duty and togetherness. In the sub-theme *3.2 Feelings as a new father*, the transition into fatherhood was mostly positive, with 61% of fathers (*n* = 8/13) reporting a smooth and joyful adjustment, whereas 38% encountered a brief period of adaptation. Fathers took pride in their new responsibilities, experiencing significant emotional growth and finding daily caregiving moments profoundly rewarding and transformative.

Emotional health, actually it got boosted up. When I heard that, I’m blessed with a baby girl in the hospital, I got so excited I started jumping, ha-ha, and I started behaving like a kid. I called all my friends and said, “I have become a father, you’re not.” Ha-ha. It boosted my mental health, and I got very much excited to see the kid. (P3)

The sub-theme *3.3 Bonding with the baby* talks about how fathers formed deep emotional bonds with their infants through daily interactions and significant milestones like first smiles, sounds, and gestures, with 69% (*n* = 9/13) reporting immense happiness from these moments. Despite their hectic schedules, 31% of fathers (*n* = 4/13) intentionally carved out time to engage in play or outings, which further strengthened their connection. *Memorable Moments* sub-theme 3.4, such as feeling the baby’s first movements—as experienced by 23% of fathers (*n* = 3/13) and being present at childbirth—in the case of 77% of fathers (*n* = 10/13) had profound effects on them. Fifty-four percent of the fathers (*n* = 7/13) valued early developmental milestones and actively captured these moments through photos or videos, reinforcing enduring emotional connections and enriching the early stages of parenthood with meaningful joys.

The first time I heard my baby’s heartbeat on the ultrasound, it was surreal. That moment is unforgettable. (P12)

### Theme 4: Adjusting to Infant Care and Responsibilities

This theme captures the emotional and practical challenges first-time fathers encounter while caring for a newborn. This theme comprises three sub-themes: *Early caregiving challenges, Infant health and paternal soothing, and Work–family balance.* Ninety-two percent of the fathers (*n* = 12/13) talked about their experiences taking care of their newborn as well as soothing and managing daily care duties. Fathers described adjusting their routines to attend to both their infant and their partner during the early months of parenthood. Their accounts highlight both the emotional demands and growing sense of responsibility involved in caring for their infant.

No one really prepares you for how challenging it is. You worry about everything, if the baby is eating enough, sleeping well, or even breathing properly at night. But amidst all the stress, there’s this deep sense of love and responsibility that keeps you going. (P7)

First-time fathers encountered considerable *Early caregiving challenges* sub-theme 4.1 during the early stages of parenthood, characterized by physical fatigue, emotional turmoil, and financial pressures. The major obstacles included coping with sleepless nights, deciphering their infant’s needs, and addressing feeding challenges, particularly 69% of fathers (*n* = 9/13) felt overwhelmed by persistent crying and 31% (*n* = 4/13) had trouble recognizing feeding signals. The financial strain from medical costs heightened their emotional distress. At the same time, fathers assumed an active role in caring for their infant’s health, with 46% of fathers (*n* = 6/13) reporting stress related to colic, frequent illnesses, and medical appointments, while 38% (*n* = 5/13) felt anguish at witnessing their child in discomfort. Despite these obstacles, 54% of fathers (*n* = 7/13) participated in comforting activities such as rocking and storytelling, gradually building their confidence in caregiving as outlined by the sub-theme *4.2 Infant health and paternal soothing*. These accounts suggest that although the initial transition to fatherhood was filled with uncertainty and emotional challenges, fathers’ proactive participation in caregiving and health management gradually increased their confidence and strengthened their bond with their child.

In the third month, I saw a technique to do it on YouTube. I saw a video where a couple, they used to do blogs . . . and I saw like how they’re calming and soothing the baby so that baby will sleep and I saw like they are keeping a pillow in the lap and they are keeping the baby, and then they were just shaking the pillows . . . it’s kind of a “Jhoola” activity, and automatically the baby started sleeping. And I followed the same thing for our baby, and she also habituated to that. So, that time what we started like, I used to, start sleeping from the 5:00 AM from the morning, and I used to wait till 5:00 AM, I used to take care by sitting, and taking the baby in the lap on top of the pillow, ha-ha, and sometimes I was just moving it . . . I used to keep awake till 5:00 AM and then I just keep her in the bed and then my wife used to take care and I used to sleep. So that was like the initial routine that time. (P1)

Forty-six percent of fathers (*n* = 6/13) reported having trouble managing their increased duties at home with their work obligations. To make sure they could provide for their families, some chose to take paternity leave, while others changed jobs or modified their work schedules. Their narratives emphasize the conflict between their professional goals and family obligations, as well as the choices they made to spend more time with their partner and child.

Work used to be my top priority, but after my child was born, everything changed. My focus shifted, and I had to make adjustments to ensure I was there for my family. (P11)

The transition to fatherhood frequently required considerable *Work–family balance* sub-theme 4.3, with 31% of fathers (*n* = 4/13) dealing with intricate leave policies and 15% of fathers (*n* = 2/13) choosing to take extended or temporary breaks to assist their partner and connect with their newborn. Some encountered unexpected job relocations, while others turned down promotions or altered their positions to stay nearer to their families, indicating a change in priorities where parenting became more important than career growth. It was also indicated that 54% of fathers (*n* = 7/13) had difficulties in juggling demanding work hours with the wish to spend quality time with their child and partner. Feelings of guilt and regret over missing important milestones were prevalent, particularly among those who worked long hours or travelled often. Despite these obstacles, numerous fathers intentionally reorganized their schedules, took additional leave, or opted for remote work to be more involved. The primary takeaway from both sub-themes is that early fatherhood prompted many men to reassess and adjust their professional lives, placing significant importance on family presence and emotional bonds rather than career advancement.

I was supposed to be transferred just as my wife’s pregnancy was progressing. It was hectic planning everything. In the month of November 2022, after 2-3 months she came to Siliguri, and I already joined my posting there, Umm, then it was like, every 14 days I will come back from my office. I’ll close my office early and I’ll come back and this will continue, I think, till March end. After that, I put up my paternity leave application in my office, so it was like a constant journey for me. (P2)

### Theme 5: Support Systems

This theme explores how fathers’ decisions and behaviours throughout pregnancy and the early stages of caregiving were greatly influenced by the practical and emotional support they got from friends, family, and experts. This theme consists of four sub-themes: *Support and reassurance for partner, Grandparents’ support, Availability of house help, and Professional and social Support.* Their experiences demonstrate how important it is to have a solid support network when managing the responsibilities of early parenthood.

Support comes in different ways. For me, it was about being there for my wife, understanding her needs, and making sure she was comfortable. That was my priority. (P1)

Fathers highlighted the vital importance of emotional and practical assistance within the family during pregnancy and the early stages of parenthood. In the sub-theme *5.1 Support and reassurance for partner*, 69% of fathers (*n* = 9/13) actively comforted and cared for their partners, taking on caregiving responsibilities, altering schedules for medical visits, and fostering a nurturing home atmosphere. They saw themselves as emotional supports, striving to alleviate both physical burdens and emotional stress. At the same time, 77% of fathers (*n* = 10/13) pointed out the crucial role of *Grandparents’ support* sub-theme 5.2, particularly grandmothers, who offered direct childcare, emotional support, and helped mothers recuperate after delivery. The presence of grandparents provided fathers with a sense of reassurance and facilitated the transition to parenthood. While some fathers needed to adapt to traditional caregiving roles or reorganize their living spaces, the overall effect of this family support was largely beneficial. In addition, the sub-theme *5.3 Availability of house help* outlined that 38% of fathers (*n* = 5/13) received assistance from domestic help (such as nannies or cooks), which relieved household responsibilities when family support was lacking, though 23% (*n* = 3/13) faced challenges without such help due to moving or financial limitations, underscoring the unequal access to external caregiving resources.

My mother came after the eighth month of pregnancy and was fully available to help my wife take care of the baby. Her presence reduced a lot of our worries. (P8)

Outside the household, fathers utilized a network of professionals, peers, and workplace resources to manage their newfound responsibilities. Sixty-nine percent of fathers (*n* = 9/13) indicated that health care providers and doctors were crucial in providing medical advice, addressing their concerns, and guaranteeing the safety of their newborns. Meanwhile, 23% (*n* = 3/13) sought out emotional support, parenting strategies, and encouragement from friends, colleagues, and community members, often finding shared experiences as valuable as professional guidance. Some fathers participated in parenting workshops or consulted with other fathers, highlighting the significance of learning from peers as emphasized by the sub-theme *5.4 Professional and social support.* Twenty-three percent of fathers (*n* = 3/13) experienced organizational assistance through paternity leave, flexible working hours, or supportive supervisors, which enabled them to be present during important early weeks. Nevertheless, the uneven availability of these benefits highlighted the necessity for improved systemic workplace support. All of these external support networks were instrumental in alleviating stress and allowing fathers to engage more fully in early caregiving.

I didn’t face any challenge because I got the support from the office and my managers and my leads, they helped me like I’ve taken leave whenever required, without inflicting any challenge. (P3)

## Discussion

This qualitative study explored the experiences of first-time fathers during the transition to parenthood, focusing on paternal concerns for maternal well-being, identity transformations, positive experiences of fatherhood, caregiving challenges, and the support systems that shaped fathers’ coping.

The findings of this research emphasize the diverse experiences of first-time fathers, highlighting their active engagement during the transition to parenthood. These findings are consistent with the work of Benzies and Magill-Evans (2015), Kowlessar et al. (2015), and Koyuncu and Kıssal (2025), who reported that fathers are increasingly participating in caregiving roles during the perinatal period. Similar to the present study, their research highlights the growing recognition of fathers as active contributors to early childcare rather than secondary caregivers, suggesting a cultural and psychological shift towards viewing fathers as emotionally engaged and competent partners in parenting.

Paternal Concerns for Maternal Well-being (Theme 1) highlighted that fathers took on the responsibility of ensuring their partners’ health, nutrition, and emotional stability during both pregnancy and postpartum period, dealing with issues like miscarriage, complications during pregnancy, challenges during delivery, and difficulties after childbirth. Fathers navigated through superstitious beliefs, with the majority preferring to rely on contemporary medical guidance.

These findings are consistent with previous research. For example, Hodgson et al. (2021) reported that first-time fathers often felt powerless and invisible in health care settings following miscarriage, reflecting the emotional vulnerability observed among some fathers in the present study. Similarly, during the prenatal period, Talley (2017) noted that fathers frequently experienced anxiety related to both their partner’s health and potential pregnancy complications. Leahy-Warren et al. (2022) highlighted that fathers often prioritize relational and emotional support for their partners during pregnancy, demonstrating commitment to caregiving roles in the prenatal phase.

Challenges during delivery described by fathers in the current study resonate with findings by Hodgson et al. (2021) who observed that fathers may experience helplessness and a lack of control during labour, particularly when they feel excluded from medical decision-making processes. Pinto et al. (2022) found that fathers who are actively involved during the prenatal phase are more likely to remain engaged after the birth of the child, suggesting a gradual shift towards more involved paternal roles.

Cultural influences on paternal involvement were evident in this study. Research by Onyeze-Joe and Godin (2020) and Premberg et al. (2011) indicated that cultural norms and traditional beliefs can shape the extent to which fathers participate in pregnancy and childbirth. While such norms were present to some extent in the current findings, many fathers prioritized modern medical advice.

Findings reflect that fathers in this study assumed active roles in supporting their partners during pregnancy and childbirth through emotional reassurance, practical assistance, and engagement with health care processes. Such active participation may contribute to stronger couple bonds and mutual trust during the transition to parenthood.

Fatherhood Transformations (Theme 2) highlighted fathers’ reflections on gender expectations, shifts in priorities and identity, mixed emotional experiences, growing responsibility, and shared parenting practices. These findings align with previous research showing that the transition to fatherhood involves psychological and lifestyle changes. Benzies and Magill-Evans (2015) reported that fathers often experience personal growth and changing perspectives as they adapt to their new role.

In relation to gender expectations, fathers in the present study reflected on the significance of their child’s gender, often balancing personal preferences, such as a desire for daughters, with family expectations for a son. Similar patterns were reported by Chawla (2024), who found that family pressures and norms regarding gender can create anxiety for new fathers in India. These findings suggest that gender expectations may still influence fathers’ experiences during the transition to parenthood.

The sub-theme of mixed feelings was reflected in prior research. Chawla (2024) again observed that fathers frequently experience both stress and happiness during early parenthood. Smith et al. (2024) found that initial worry and fear often evolved into confidence and joy after the child’s arrival. Even after positive birth experiences, fathers may continue to experience moments of doubt and emotional strain, reflecting a complex emotional experience in which love and pride coexist with insecurity and self-doubt (Škvařil & Presslerová, 2024; Vidinha et al., 2025). This supports the present study’s observation that fathers navigate both positive and uncertain emotions during this transition.

The sub-theme of paternal evolution reflects the personal growth reported in the present study. Benzies and Magill-Evans (2015) found that fathers often viewed parenthood as a catalyst for self-development, reporting changes in their priorities and perspectives following the birth of their child. Similarly, Henwood and Procter (2003) noted that fathers described gaining a stronger sense of identity and purpose through parenthood. Fathers in the present study described increased maturity and a re-evaluation of their roles and responsibilities.

Finally, the sub-theme of shared parental experiences and responsibilities aligns with research highlighting the importance of collaborative parenting. Kowlessar et al. (2015) emphasized that co-parenting strategies help distribute caregiving responsibilities and strengthen partnership dynamics. Koyuncu and Kıssal (2025) reported that although fathers may initially perceive their role as primarily financial during pregnancy, many transition into more active caregiving roles following the child’s birth. This aligns with the findings of the present study, where fathers described shared parenting responsibilities within the couple relationship.

These findings suggest that the transition to fatherhood involves ongoing emotional and personal development, with fatherhood acting as a developmental milestone that fosters emotional maturity, empathy, and greater involvement in family life.

Understanding this identity shift may be useful for mental health professionals, as it highlights both the challenges and adaptive potential of fatherhood and underscores the need to support fathers as they navigate evolving roles.

Positive Experiences of Fatherhood (Theme 3) indicated the emotional satisfaction, connection, and pride fathers experienced during pregnancy, childbirth, and early caregiving. Fathers described moments of joy, bonding, and meaning that reinforced their emerging paternal identity. These findings align with the work of Henwood and Procter (2003) and Shorey and Ang (2019), who emphasized that fatherhood can be a deeply fulfilling experience characterized by emotional closeness and engagement with the child. This perspective contrasts with research by Baldwin and Bick (2018) and Dallos and Nokes (2011), which reported challenges related to bonding and paternal identity during the transition to parenthood. Positive experiences observed in the present study suggest that early involvement may facilitate stronger emotional connections and more adaptability to fatherhood.

Upon hearing the news, many fathers expressed joy, while others described a growing sense of responsibility and anticipation for the child’s arrival. These findings are consistent with Talley (2017), who noted that expectant fathers often experience feelings of anticipation and joy. Many fathers described particularly meaningful emotional experiences following the birth of their child. This finding aligns with Henwood and Procter (2003), who observed that fathers derive satisfaction not only from spending time with their children but also from the enhanced sense of identity associated with parenthood.

Bonding with the baby emerged as a central aspect of positive fatherhood experiences. Fathers in this study reported that interacting with their infants, observing their early expressions, and participating in caregiving activities strengthened their emotional connection. Most fathers expressed immense joy in witnessing their baby’s first smiles, sounds, and gestures, which they viewed as significant moments in developing attachment. These findings are consistent with Shorey and Ang (2019), whose meta-synthesis highlighted that active interaction and partner support play key roles in strengthening father–infant relationships. Pinto et al. (2022) reported that greater involvement in infant care is associated with stronger emotional bonding, while Koyuncu and Kıssal (2025) suggested that bonding acts both as a motivation for and an outcome of developing paternal roles.

Fathers described several memorable milestones that contributed to their positive experiences of fatherhood. For some participants, prenatal experiences such as observing their baby during ultrasound scans were meaningful, birth of the child was the most significant milestone, with other fathers recalling it as an intensely emotional and unforgettable event. Half the participants reported actively documenting early developmental milestones, such as first giggles, words, and expressions, through photos and videos, reflecting their desire to preserve these meaningful moments. These findings align with Benzies and Magill-Evans (2015), who found that fathers often describe parenthood as one of the most rewarding experiences of their lives, referring to it as the “best job in the world.”

Positive engagement in caregiving, early bonding opportunities, and shared parenting experiences appear to strengthen fathers’ connection with their child. These results highlight the importance of recognizing fathers not only in terms of challenges during the transition to parenthood but also in relation to rewarding and growth-promoting aspects of fatherhood.

Adjusting to Infant Care and Responsibilities (Theme 4) highlighted the practical and emotional difficulties fathers encountered during the early stages of caregiving, including fatigue, sleep disruption, infant health concerns, and balancing work responsibilities with family life. Despite these challenges, fathers demonstrated adaptive strategies and a willingness to learn caregiving skills, reflecting increasing involvement in early parenting. These findings align with studies showing that fathers often experience initial caregiving challenges but gradually develop competence through active participation (Ganapathy, 2016; Machin, 2015; Pinto et al., 2022).

The early days after childbirth were described as particularly demanding, with fathers reporting exhaustion, uncertainty in handling the newborn, and difficulty understanding feeding routines and infant cues. Persistent crying and sleep disruptions were common sources of stress described. Similar experiences were reported by Ganapathy (2016), who noted that emotional pressures during early fatherhood can affect fathers’ sense of readiness and well-being.

Infant health concerns such as colic, sleep regression, and recurrent illnesses further contributed to fathers’ worries. In response, many fathers adopted active soothing strategies including rocking, walking, storytelling, and seeking guidance through online resources. This supports findings by Pinto et al. (2022) and Koyuncu and Kıssal (2025), who found that hands-on caregiving can strengthen father–infant bonding and enhance paternal confidence.

Work–family balance further shaped fathers’ experiences. Some fathers navigated limited leave policies or modified work commitments to spend time with their newborns. Long working hours often reduced opportunities to bond with the infant and support their partner, sometimes leading to feelings of guilt. Previous studies similarly highlight how work demands and provider expectations can restrict fathers’ involvement in early caregiving (Hodgson et al., 2023; Shorey & Ang, 2019). Flexible work arrangements have been identified as an important factor that may support greater paternal engagement (Barclay & Lupton, 1999; Henwood & Procter, 2003).

It suggests that early fatherhood involves navigating caregiving demands alongside professional responsibilities, requiring fathers to adapt to new roles while developing confidence in their parenting abilities.

Support Systems (Theme 5) highlighted the important role of relational, familial, and professional support in helping fathers adjust to early parenthood. Fathers frequently described supporting their partners through emotional reassurance, attending medical appointments, and assisting with household responsibilities. These findings are consistent with research suggesting that fathers often take on supportive roles during the transition to parenthood (Leahy-Warren et al., 2022; Pinto et al., 2022). However, previous studies also indicate that the arrival of a baby can place strain on couple relationships, reflecting the complexity of this period (Baldwin & Bick, 2018).

Family support, particularly from grandparents, emerged as a key factor easing the transition to fatherhood. Many fathers relied on parents or in-laws for childcare guidance and assistance, reflecting the importance of intergenerational support described by Onyeze-Joe and Godin (2020). The presence of domestic help also helped families manage household responsibilities, allowing parents to focus more on infant care, consistent with findings by Smith et al. (2024).

Professional and social support further contributed to fathers’ confidence and coping. Participants reported seeking advice from health care professionals, peers, and parenting resources, which aligns with research emphasizing the value of inclusive health care environments for fathers (Smith et al., 2024). While some studies suggest that fathers may feel marginalized in health care systems traditionally oriented towards mothers (Vidinha et al., 2025), the present findings indicate that supportive networks can significantly ease fathers’ path to early parenthood.

This research provides fresh and insightful perspectives on the experiences of first-time fathers, demonstrating a largely positive environment of support and engagement. In contrast to previous studies that emphasize the difficulties fathers face in obtaining assistance due to societal expectations and systemic challenges (Darwin et al., 2017; Koyuncu & Kıssal, 2025; Reay et al., 2024), the individuals in this study indicated that they benefitted from robust social networks including family, friends, health care professionals, and supportive workplace policies which facilitated their accommodation to parenthood and alleviated stress through collaborative caregiving tasks. This discovery is significant as it indicates how conducive environments may contribute to fathers’ engagement and confidence during this crucial period. These insights can guide interventions aimed at strengthening family-centred support systems in the early stages of infancy.

An interesting observation in the present study was the practical advice these fathers shared with their peers, addressing issues such as financial management and health care choices, as well as emotional preparedness and the significance of prioritizing the mother’s health. Fathers highlighted making informed decisions, dismissing outdated childcare customs, and setting healthy limits within extended family relationships, perspectives that are still insufficiently explored in current literature. This peer-driven knowledge may reflect how fathers informally exchange experiences and coping strategies during the transition period.

In contrast to much of the previous research highlighting the psychological burden of adhering to traditional gender roles and societal norms (Dallos & Nokes, 2011; Reay et al., 2024; Škvařil & Presslerová, 2024; Vidinha et al., 2025), the fathers participating in this study exhibited a flexible, family-oriented approach. They accepted responsibilities for caregiving and household tasks based on what was best for their partner and child, often ignoring superstitions and strict norms. Such adaptability may reflect evolving paternal roles in which nurturing, emotional openness, and collaboration are increasingly valued. This change may suggest a wider cultural shift towards a more emotionally open and involved form of fatherhood, bolstered by strong social networks that may mitigate the adverse effects of societal pressures.

Ultimately, the experiences of these fathers illustrate that fatherhood transcends a simple role; it represents a meaningful and evolving relationship defined by resilience, affection, and active engagement. Their hands-on participation is associated with enhanced mental well-being and stronger family ties, emphasizing the crucial importance of emotional investment and joint caregiving in nurturing a cohesive family atmosphere (Vidinha et al., 2025). This finding emphasizes the potential value of father-inclusive practices in perinatal and infant care are essential rather than optional. This research deepens our understanding of early fatherhood by showcasing advancements in paternal involvement despite the potential influence of broader gender norms on paternal experiences in various cultural contexts. Although many fathers in this study reported receiving strong support from family and professional networks, the findings highlight the potential value of continuing to include fathers in prenatal and postnatal care settings. Encouraging father participation and providing guidance where needed may further strengthen family functioning during the transition to parenthood.

### Limitations, Implications, and Future Recommendations

This study provides important insights but has several limitations. The sample consisted primarily of urban, English-speaking fathers, which limited cultural and linguistic diversity. As a result, the findings may not reflect the experiences of fathers from rural settings or those who do not communicate in English.

An additional limitation is that all participants were highly educated. Fathers with higher levels of education and socioeconomic resources may be more likely to endorse egalitarian views and participate actively in caregiving. In particular, the apparent shifts in gender roles and paternal involvement observed in this study should be interpreted with caution, as they may reflect the attitudes and opportunities available to more privileged groups.

Despite these limitations, this study highlights the emotional and caregiving contributions of first-time fathers in India and suggests the potential value of supportive policies that recognize fathers’ roles during the transition to parenthood. Greater attention to paternal mental health, inclusive health care practices, and policies that support shared parenting may benefit families during this period. Health care professionals may consider adopting more father-inclusive approaches, such as involving fathers during prenatal care, offering couple-based support, and acknowledging paternal stress. Workplace policies such as paternity leave and flexible work arrangements may facilitate paternal involvement and support fathers’ emotional well-being during early parenthood.

Future research should involve a more diverse range of participants, including non-English speakers, fathers from rural communities, non-biological fathers, and individuals from varied socioeconomic and educational backgrounds. Exploring workplace policies and structural supports may provide further insight into factors that shape paternal involvement. Longitudinal research could monitor the changes in paternal roles over time, providing essential understanding of how fathers’ emotional, relational, and caregiving abilities evolve as their children mature and how societal and institutional elements continue to influence these developments.

## Conclusion

This qualitative study explores the emotional journeys of first-time fathers during infancy, highlighting both their struggles and moments of joy. It reveals a shift from traditional gender roles, with fathers actively engaging in caregiving and emotional support. Strong support systems including family, health care, and workplace policies aided this transition. The findings emphasize the need for inclusive recognition and support for all fathers, regardless of background.

## Supplemental Material

sj-docx-1-jmh-10.1177_15579883261445266 – Supplemental material for Exploring the Emotional Experiences of First-Time Fathers During Infancy: A Qualitative Study Supplemental material, sj-docx-1-jmh-10.1177_15579883261445266 for Exploring the Emotional Experiences of First-Time Fathers During Infancy: A Qualitative Study by Sharanya Chatterjee, Vaishalee Saravanan and Samyukta Vaidyanathan in American Journal of Men's Health

sj-docx-2-jmh-10.1177_15579883261445266 – Supplemental material for Exploring the Emotional Experiences of First-Time Fathers During Infancy: A Qualitative Study Supplemental material, sj-docx-2-jmh-10.1177_15579883261445266 for Exploring the Emotional Experiences of First-Time Fathers During Infancy: A Qualitative Study by Sharanya Chatterjee, Vaishalee Saravanan and Samyukta Vaidyanathan in American Journal of Men's Health
