# Supplementary material for: Exploring the Emotional Experiences of First-Time Fathers During Infancy: A Qualitative Study
Source: Am J Mens Health. 2026 May 23;20(3):15579883261445266. doi: 10.1177/15579883261445266 (PMC13199690; doi:10.1177/15579883261445266)
Supplement: sj-docx-1-jmh-10.1177_15579883261445266 – Supplemental material for Exploring the Emotional Experiences of First-Time Fathers During Infancy: A Qualitative Study [file sj-docx-1-jmh-10.1177_15579883261445266.docx]

**Clinical Data Sheet**

This will include the following details:

● Mode of Pregnancy

i) Natural Conception

-Yes/No

ii) Assisted Conception

-Yes/No

-If yes, whether:

a) Ovulation Induction (OI)

b) In Vitro Fertilisation (IVF)

c) Intrauterine Insemination (IUI), or

d) Intracytoplasmic Sperm Injection (ICSI).

- Any history of pregnancy loss in the past?

-Yes/No

● Mode of delivery:

A. Normal Delivery

-Yes/No

B. C -Section

-Yes/No

-If yes, whether:

a) Elective or

b) Emergency.

● Presence of any gestational complications:

-Yes/No,

-if yes, please mention-

- Was the baby born premature?

-Yes/No

- Birth weight of the child -

● Presence of any physical health issues in the child:

-Yes/No,

-if yes, please mention-
