# Supplementary material for: Exploring the Emotional Experiences of First-Time Fathers During Infancy: A Qualitative Study
Source: Am J Mens Health. 2026 May 23;20(3):15579883261445266. doi: 10.1177/15579883261445266 (PMC13199690; doi:10.1177/15579883261445266)
Supplement: sj-docx-2-jmh-10.1177_15579883261445266 – Supplemental material for Exploring the Emotional Experiences of First-Time Fathers During Infancy: A Qualitative Study [file sj-docx-2-jmh-10.1177_15579883261445266.docx]

**Validated Semi – Structured Interview Schedule**

*(Validated by 5 experts: licensed clinical psychologist, psychiatrist, neonatologist, perinatal mental health specialist, and qualitative research professional)*

**1. What were the initial emotions or thoughts that were prominent for you when you first found out you were going to be a father?**

*a. What was the period of pregnancy like for you as a first-time father?*

*b. Could you elaborate on your thoughts and feelings leading to your decision to be a father?*

*c. What were the initial emotions expressed by your close family members?*

**2. In what ways did you anticipate that your life will change after the baby arrived?**

*a. How did you anticipate your role as a father, evolving?*

*b. Were there any changes you noticed in yourself after becoming a father?*

*c. How did you prepare for those changes?*

**3. Can you tell me whether you accompanied your wife for ultrasounds during pregnancy?**

*a. If yes, how was your overall experience as a first-time father?*

*b. Can you describe how the early stages of fatherhood was like for you?*

*c. Can you tell me about a typical day in your life as a first- time father?*

**4. From your experience, were there any specific challenges you faced as a first-time father?**

*a. If no, what makes you say so?*

*b. If yes, can you provide some examples of the types of challenges you faced during the stages of* *pregnancy and post childbirth?*

*c. How did you work through* *them in the process?*

**5. As a first-time father, were there any particular times or incidents that had a negative impact on your emotional health?**

*a. If no, how was your emotional health at that time?*

*a. If yes, please tell me more.*

**6. In the process, did you seek or desire any specific support?**

*a. If no, can you tell me why?*

*b. If yes, please elaborate.*

*c. Were there any barriers you encountered in receiving that support?*

*d. How did you overcome those barriers in receiving that support?*

**7. Are there any kind of common beliefs, stereotypes, or expectations about “fatherhood” prevailing in our society according to you?**  *a. If no, what is your idea about “fatherhood”?*

1. *If yes, did it have any kind of impact on your mental health and overall wellbeing?*

*c. Was there any pressure to live up to certain expectations society had of parenthood and if yes, how did you handle it?*

**8. Can you elaborate on the psychological consequences, if any, of the gender roles or stereotypes associated with "fatherhood”?**

*a. How did you personally cope with or challenge any gender roles/stereotypes associated with fatherhood that may have influenced your emotional* *experiences?*

**9. Were there any memorable positive experiences you had during your journey, as a first-time father?**

*a. Can you share some of those positive experience that brought you joy or a sense of fulfillment during this time?*

**9. Looking back, what insights or advice would you offer to other fathers going through a similar experience?**
